# Effects of marbling on physical and sensory characteristics of ribeye steaks from four different cattle breeds

**DOI:** 10.5713/ajas.20.0201

**Published:** 2020-06-24

**Authors:** Nurul Nuraliya Shahrai, Abdul Salam Babji, Mohamad Yusof Maskat, Ahmad Faisal Razali, Salma Mohamad Yusop

**Affiliations:** 1 Department of Food Sciences, Faculty of Science and Technology, Universiti Kebangsaan Malaysia, 43600 UKM Bangi, Selangor, Malaysia; 2 Karnivormalaya, Gourmet Artisan Enterprise, Bukit Jelutong Gate, 40150 Shah Alam, Selangor, Malaysia

**Keywords:** Marbling, Beef, Ribeye Steak, Texture, Quantitative Descriptive Analysis

## Abstract

**Objective:**

Marbling or intramuscular fat (IMF) has been widely reported to directly impact the sensory acceptance of meat. This study was carried out to determine the physical and sensory characteristics of ribeye, *Longissimus dorsi* steaks obtained from four different cattle breeds namely Wagyu, Angus, Brahman, and Malaysian local beef, the Kedah-Kelantan (KK).

**Methods:**

The degree of marbling was determined by using an established combined camera-image analysis technique while instrumental texture determination was carried out by using Warner-Bratzler shear force analysis. Sensory evaluation of the beef steaks was performed following a quantitative descriptive analysis incorporating 10 trained consumer panelists.

**Results:**

Wagyu was found to possess the highest (p<0.05) percentage of IMF at 33.90% and the lowest shear force (raw = 5.61 N/mm^2^; cooked = 14.72 N/mm^2^) followed by Angus (20.87%), Brahman (12.17%), and KK (p<0.05, 6.86%). The difference in sensory properties of the four steaks was evident, with Wagyu appearing to be highly correlated with most sensory attributes measured namely sustained buttery, tooth-packing, chewiness, juiciness, tenderness, mouthfeel, oiliness, and overall acceptability. The Malaysian local beef, KK was found to be less acceptable (p<0.05), although most of its sensory attributes were found similar (p>0.05) in appearance, aroma, texture, juiciness, and flavour to the cooked steak from Angus and Brahman.

**Conclusion:**

This present study demonstrated the role of IMF in determining the quality and sensory acceptance of beef from different cattle breeds. These data have provided new information and further understanding on the physical and sensory quality of Malaysian local beef.

## INTRODUCTION

Ribeye-steak is an incredibly popular category of meat products, well-known for its high marbling and prepared generally by fast and hot cooking method such as searing, pan roasting, or grilling. According to America Meat Science Association 2015 (AMSA 2015) [1], ribeye steak is taken from the 6th until 13th rib and is mostly composed of *Longissimus dorsi* (LD) muscle. The palatability of ribeye steak is varied according to the types of animal and species. It can be identified by its marbling and assessment of textural instrumental analysis such as Warner Bratzler shear force (WBSF) and human panel assessors [2,3]. Ribeye marbling is one of the purchasing factors which may lead to beef palatability [4,5].

Marbling assessment is widely established in beef manufacturing countries such as USA, Japan, Australia, and Korea. Computer image analysis was implemented by Japanese Meat Grading Association and is considered as one of the best methods in determining marbling score [6,7]. The first information about a quality steak can be determined by its visual appearance, which shows positive correlation with sensory acceptability and textural quality of the meat [3,6,7]. Kuchida et al [8] quantified the marbling area ratio in the LD surface by image processing. Since then, computer vision has been extensively studied and considered as the most potential technique for grading of beef marbling due to its best match to human eyes and the combination of non-destructiveness, speediness, and simplicity in sample preparation [3,8].

Development of reliable statistical model derived from human panels with varied marbling levels, has been proven to be useful to the beef industry. Although the instrumental analysis is used, the human panel is believed as an imprecise predictor to produce more standardised responses. According to previous work, the role of intramuscular fat (IMF) can be clearly observed as the increase of marbling degree clearly improves the sensory scores of tenderness, juiciness and beefy flavour, and steak palatability [3,4,9–12]. However, Lee and Choi [13] reported a greater correlation between flavour and overall acceptability of beef than juiciness or tenderness.

Many studies have been carried out on the effects of marbling on IMF and meat quality in many cattle breeds, including Wagyu [6,7,14], Angus [15], and Brahman [16]. However, the IMF level and meat quality acceptance of Malaysian local cattle, Kedah-Kelantan (KK) have been scarcely documented. In Malaysia, KK is categorised as *Bos indicus* breed, prevails as Malaysian beef cattle, in which the production is often applied within inconsistent feeding regimes and lack of proper management [17]. However, this breed of cattle is popular due to high fertility, immunity, and resistance to heat stress. Therefore, the objective of this study is to compare the physical and sensory characteristics of ribeye steaks from different cattle breeds namely Wagyu and Angus (*Bos taurus* group), as well as Brahman and KK (*Bos indicus* group). The correlation between marbling and sensory attributes of these beef samples was also evaluated by using Partial least square regression (PLSR).

## MATERIALS AND METHODS

### Preparation of samples

Frozen block samples of ribeye steaks of different breeds (Wagyu, Angus, Brahman, and KK) were packed under vacuum and transported in an ice box (below 4°C) from a local meat supplier, Gourmet Artisan Enterprise, Bukit Jelutong, Malaysia. The samples were cut into 1.5-cm thickness, following the guidelines outlined by AMSA [1]. The sliced samples were kept in the freezer (Hitachi Ltd, Tokyo, Japan) at −20°C until further analysis. The samples were thawed at 5°C overnight prior to cooking.

### Determination of the marbling level and size of ribeye steak

The lighting box was built according to Basset et al [18] with slight modification. The length and width of each steak sample were measured by using a measuring tape. The image of ribeye steak was captured by a high resolution camera (ZB601KL, Android 8.1-Oreo, Guangzhou, China) and was subsequently uploaded into Image-J Software (Image-J.net, Madison, WI, USA) for marbling degree determination. Principally, the Image-J Software differentiates the IMF and lean muscles through the percentage of white to the black contour of meat, respectively.

### Warner-Bratzler shear force determination

Steaks designated for instrumental analysis were removed from freezer storage and allowed to thaw for 24 hours at 5°C in a chiller (Hitachi Ltd, Japan). Steaks were cooked at 190°C to 232°C and cooled at room temperature of 23°C for 2 min [1]. The steaks were cut approximately 1.5 cm^3^ for texture analysis. Similar size of raw samples was used as comparison. The samples were analysed by touching the load at the parallel to longitudinal axis of sample, using a TA-XT2 texture analyser (Texture Technologies Corp., Stable Micro Systems, Godalming, UK) with 1-mm thick of Warner-Bratzler shear blade. The shear force was defined by peak force during first compression cycle as outlined by several studies [19,20].

### Sensory evaluation

#### Sample preparation

The ribeye steak samples were thawed overnight at 5°C and cooked by using an open air broiler (Chelstar, CGC-411_YD933/8050, Penang, Malaysia) at a temperature between 190°C and 200°C following the recommended protocol by AMSA [1]. The temperature was monitored by using an infrared thermometer (GS320, Shenzen, China). Samples were cooked at specific doneness (medium-well) until reaching an internal temperature of 71°C±1°C.

For sensory evaluation, the cooked ribeye steaks were cut into approximately 1.5 cm×1.5 cm cubes and wrapped with aluminium foil [1]. The prepared samples were placed in an oven to keep the sample in ideal serving temperature of 68°C [1,9]. The descriptive sensory profile of four ribeye steak samples was established by using quantitative descriptive analysis (QDA) as reported by O’Sullivan [2,21–23].

### Quantitative descriptive analysis

#### Lexicon development

Four expert panellists from the Department of Food Sciences, Faculty of Science and Technology, Universiti Kebangsaan Malaysia (UKM) participated in the lexicon development. Each panellist had completed at least 120 min of general descriptive sensory testing, including food products with descriptors similar to those that might be found in beef. Initially, the panellists evaluated each beef sample in groups and wrote down the list of descriptors present in the sample. Then, the panel leader led a discussion to reach an agreement on the colour, texture, flavour, and aroma descriptors present in the sample. After the group discussion, a consensus was reached and 26 key attributes with their respective definitions were selected. The descriptive attributes and definitions (Table 2) which include appearance, texture, juiciness, aroma- and flavour-related attributes, after-taste, and overall acceptability were added along with other descriptors to judge the extent of liking of the product [2,20]. The screening steps of these attributes were also referred and compared with a series of meat palatability research carried out by O’Sullivan et al [2,22–25].

#### Training and selection of assessors

Prospective members of the descriptive panel were recruited among a pool of undergraduates from the Faculty of Science and Technology in UKM. Pre-screening questionnaires [24] were distributed to them prior to the screening process. The prospective panellists were then subjected to a series of screening tests. The QDA test was conducted according to the method expressed by O’Sullivan [2]. A 10-member sensory panel (five females and five males, aged around 22 to 24 years) was recruited. The panellists were trained in QDA techniques in three 2-hour training sessions. All panellists were pre-screened for the ability to differentiate samples by using reliability test (Table 1). Those who could not detect the intensity of low to high concentration of sweetness, saltiness, and sourness were excluded.

#### Familiarisation process

On the first day of training session, an overview of QDA was briefed to the participants. A ranking test, following a method by Meilgaard et al [24] was carried out to determine the candidate’s ability to discriminate grading levels of intensity of a given attribute. On the subsequent day, a training sample was cooked, coded, and presented to the group of which they were asked to evaluate by using the outlined sensory terms. Two 1.5-hour training sessions were carried out in order to familiarise the panel with varying intensity of sensory attributes of ribeye steak (Table 2). For the remaining of the training sessions, the panellists were exposed to an informal pretest where control samples (steak cubes) were presented as warm-up samples.

#### Actual testing

Actual testing was prepared as suggested by O’Sullivan [2] and American Meat Science Association [1]. The steaks were cut at approximately 1 cm^3^ of size. They were wrapped in aluminium foil to prevent cooking loss, aroma loss, and kept in an oven (Universal Oven UN30plus, Memmert, Schwabach, Germany). The cooked samples were wrapped in the aluminium foil and stored in a heated oven with the temperature of 68°C prior to serving. The sensory scale used was a 150-mm line scales anchored from left to right with the term ‘none’ to ‘strong’. The responses were recorded by measuring the distance in mm (1 to 150 mm). The quantitative descriptive sensory evaluation was carried out by 10 semi-trained panellists.

The panellists were provided with a warmed sample in an individual sealed vial, toothpick, serviette, palate cleansers (a cup of plain water, unsalted cracker), and olfactory flusher (a vial of coffee bean) in order to reduce the carry-over effect from previous samples. Each panellist received a set of sample ballots (4 sheets) and the served vial was labelled with three random digits. The purpose of using random digit is to eliminate bias due to the order of presentation [2,26–28]. Before the sensory evaluation started, panellists were given verbal instructions about each attribute in the sensory ballots.

### Statistical analysis

The effects of different cattle breeds on marbling, physical, and sensory characteristics of ribeye steak were analysed using analysis of variance (one-way ANOVA) to determine the existence of statistical difference (p<0.05), followed by DUNCAN test to determine the statistical difference among the means (IBM SPSS Statistic 20). The spatial relationships of these properties were summarised using ANOVA-partial least squares regression (APLSR) employing the XL-STAT software (Addinsoft Inc., Brooklyn, NY, USA) to analyse the descriptive and raw instrumental data. Results from all the tests were expressed as mean±standard error.

## RESULTS AND DISCUSSION

### Intramuscular fat level and the size of ribeye muscles

**Figure 1:**
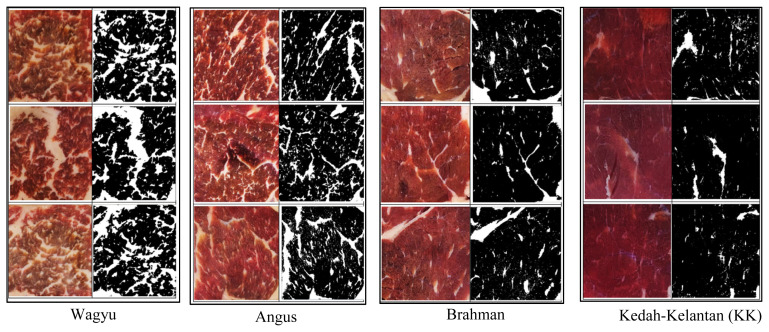
Intramuscular (IMF) view of ribeye steak from four different cattle breeds.

Figure 1 shows the IMF view of ribeye steak from four different cattle breeds with Table 3 contains information about the physical characteristics of the samples. The type of breed was found to significantly affect (p<0.05) the percentage of IMF and the physical characteristics of meat samples. The size of Wagyu ribeye steak was the biggest (p<0.05) among all samples, with 17.40 cm in length and 11.43 cm in width. This is followed by Angus, Brahman, and KK, respectively. The smallest size (p<0.05) of ribeye steak was recorded by the Malaysian local cattle (KK), as justified by the small body conformation which is in the range of 430 to 460 kg for bull and 220 to 250 kg for female [17]. This finding confirmed the influence of breed type on the size of ribeye steak as it is in agreement with many previous studies related to the effect of size and weight of live animals on carcass and muscle size [5].

In general, IMF has a positive effect on beef tenderness, juiciness, flavour, and overall acceptability as all these attributes score higher with increasing level of IMF [2,3,11,13]. Conventionally, marbling of meat is assessed by visual appraisal or chemical analysis, which has the disadvantages of being subjective and time-consuming. In this study, a computer-camera image analysis was performed to segment marbling from lean LD muscle. Based on reflectance characteristics, the IMF (white contour) was distinguished from lean muscle (black contour) and generated binary muscle images (Figure 1). It was found that raw Wagyu had significantly (p<0.05) the highest IMF (33.90%), followed by Angus (20.87%), Brahman (12.17%), and KK (6.86%). These results suggested the variation of IMF concentration in different types of cattle breed. Previous studies underlined that several factors may influence IMF level which include types of cattle breed [5–7], cattle groups such as *Bos taurus* and *Bos indicus* [29,30], muscle position in the carcass [5], or genetic inheritance [5,16].

The WBSF results, as reported in Table 3 suggested that marbling substantially improves the tenderness of ribeye steak. The highly marbled Wagyu beef was extremely tender, followed by Angus, KK, and Brahman. These data support the findings from Cheng et al [4,6,7,10] on the relationship of IMF with beef tenderness. Rodrigues et al [16] reported that higher shear force is relative to the degree of hardness and associates well with *Bos indicus* cattle, which is tougher and leaner (p<0.05) than meat from *Bos taurus* cattle, regardless of the marbling score. The role of IMF, according to Lee and Choi [13], includes the weakening of structural integrity in muscles, preventing the muscle fibre from forming cross-links and enabling the muscle to be broken up more readily in the mouth.

### Quantitative descriptive analysis

Table 4 displays the QDA results of ribeye steak from four different cattle breeds. There were significant differences between samples for three attributes (out of 26 sensory traits) which include oiliness, tenderness, and overall palatability. There were no differences (p>0.05) in terms of nutty, sweet, sour, and beefy aroma among all samples. Wagyu exhibited the lowest (p<0.05) intensity of chewiness and tooth-packing while KK was observed (p<0.05) to be the most bitter in flavour. Brahman was also found to be very highly chewable (p<0.05) as compared to Angus, Wagyu, and KK but no difference (p>0.05) was observed in terms of tooth-packing intensity among samples.

A relatively high intensity rating was consistently observed in Wagyu in terms of surface colour, internal colour, texture and grain, sour, beefy, texture oiliness, juiciness, mouthfeel, salty flavour, sweet flavour, sour flavour, umami flavour, beefy flavour; Beefy, roasted flavour, overall flavour impact, sustain buttery, and overall acceptability attributes (Table 4). Nevertheless, there was an insignificant difference (p>0.05) observed between Wagyu, Angus, Brahman, and KK except in oiliness, tenderness, and overall palatability. The presence of IMF and high fat content in Wagyu contributed to the oiliness, tenderness, and satisfaction of juiciness and aftertaste. In a recent study on sensory acceptability of grilled beef samples, Frank et al [3] and Corbin et al [9] noticed that most flavour-related attributes are also significantly correlated with the level of IMF. Our findings suggested that the importance of marbling (IMF) in beef samples does not only rely on the fact that fat may carry inert flavour profile and give the samples’ distinctive taste, but also possibly contribute to the succulency or ability of meat to retain its juice after cooking.

According to Table 4, there was no significant difference (p>0.05) in aroma-related attributes and most flavour-related attributes except for bitterness, roasted flavor, and overall flavour acceptance as perceived by trained panellists. However, KK can be clearly distinguished from the rest of the samples based on its high bitter intensity (p<0.05) which may explain its poor overall flavour intensity (OFI) likings. Two possible factors that may cause higher bitterness in KK are Maillard reaction and burning of haemoglobin. Sometimes, burnt and roasted as positive flavour attributes described as caramelisation are developed in a steak by dry cooking method which appeals to the human senses. However, burning is not suitable for high lean meat that has less IMF such as KK ribeye steak as it will be more profound within dark firm dry (DFD) meat. This bitter flavour is developed due to the Maillard reaction [3] as it is known in combination of carbonyl group (sugar) and amino acid (protein), forming an unsightly brown compound which is responsible for the undesirable bitter taste. Moreover, high lean meat is believed to have high myoglobin, or high heme group.

According to Frank et al [3], IMF might contribute to the perception of sweetness, umami, saltiness, and sourness in beef samples. This study showed otherwise, as there were not much differences in Wagyu, Angus, Brahman, and KK beef samples for most specific aroma- and flavour-related attributes measured. Nevertheless, the OFI likings (Wagyu>Angus> Brahman>KK) did imply the significance of IMF contribution.

Regardless of the cooking method, ribeye steak colour will gradually deteriorate where its internal and external surface will change as cooking progresses due to heat affecting the haemoglobin pigment and organic compound of meat. The ideal resultant cooked steak colour possesses certain browning on the surface and persistent pink on the internal. According to Frank et al [3], a heme-chrome pigment of beef after cooking is produced by denaturation of globin and heme iron and thus, this heme-chrome pigment cannot revert to a red pigment. The change in steak colour from red (raw) to brown (cooked) is due to Maillard reaction and caramelisation which are also responsible for the development of the unique flavour and steak colour. The high intensity of after-taste, specifically of sustained buttery attribute was evident (p<0.05) in Wagyu which explained the positive contribution of IMF in making this sample highly accepted by the semi-trained panellists than the rest of the samples.

### Relationship between sensory descriptive analysis, intramuscular fat content, and cattle breed

**Figure 2:**
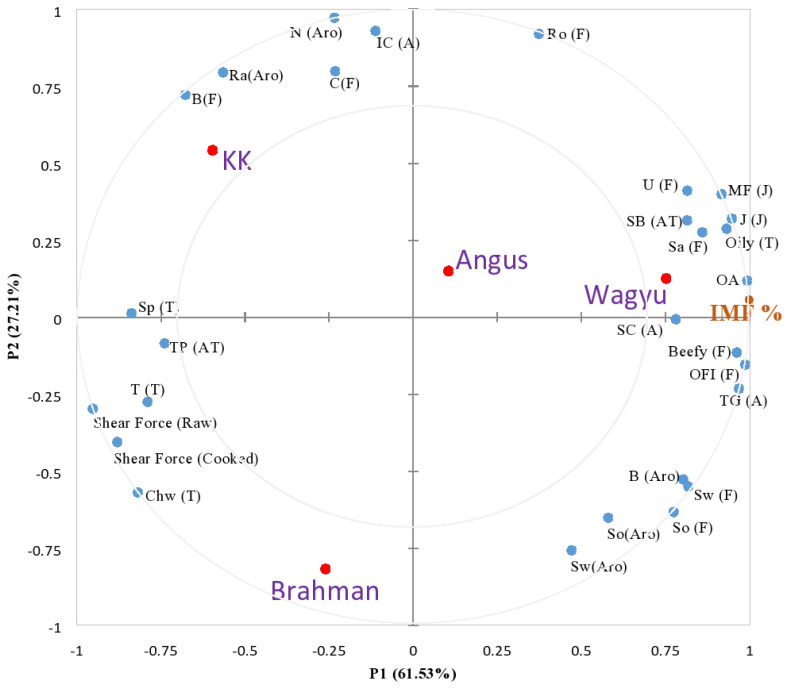
Overview of the variation found in the data from the analysis of variance partial least square regression (A-PLSR) correlation loadings plot for the intramuscular fat (IMF) and sensory characteristics of cooked meat samples from four different cattle breeds (Wagyu, Angus, Brahman, and Kedah-Kelantan, KK. Surface Colour-SC (A), Internal Colour- IC (A), Texture and Grain-TG (A), Sweet aroma-Sw (Aro), Sour Aroma-So (Aro), Beefy Aroma-B (Aro), Nutty Aroma-N (Aro), Rancid Aroma-Ra (Aro), Oiliness-Oil (T), Springiness-Sp (T), Chewiness-Chw (T), Tenderness- T (T), Juiciness- J (J), Mouthfeel-MF (J), Salty-Sa (F), Sweet-Sw (F), Sour-So (F), Bitter-B (F), Umami-U (F), Caramel-C (F), Beefy-Beefy (F), Roasted-Ro (F), Overall Flavour Impact-OFI (F), Sustained Buttery-SB (AT), Tooth-packing- TP (AT), and Overall Acceptance-OA).

Figure 2 elucidates the relationships among the descriptive sensory data and factors including different cattle breed (Wagyu, Angus, Brahman, KK) and IMF content through an APLSR correlation loadings plot. It is evident that most steak sensorial attributes are located close to the upper right quadrant and adjacent to the Wagyu sample, which include mouth feel, juiciness, oily texture, tenderness, sustained buttery of after taste, beefy aroma, umami flavour, roasted flavour, and overall flavour intensity. This observation clearly distinguished the sensory quality of Wagyu from the rest of the samples, which particularly explained the overall acceptability of Wagyu, highly associated with myriad unique-intense beefy flavours, juiciness, and succulence as contributed by the high IMF (33.90%).

The location of Angus being adjacent to Wagyu as compared to KK and Brahman also implies certain similarities of sensory characteristics between Wagyu and Angus, which can be observed in the QDA results (Table 4). Angus is plotted on the right quadrant, and correlated well with most of the sensory attributes which strongly characterise Wagyu, including IMF. Wagyu and Angus, both coming from *Bos taurus* group possessed among the highest in IMF with Wagyu scoring 33.90% and Angus scoring 20.87%. The high concentration of marbling by IMF clearly contributed to a positive correlation with mouth feel, juiciness, tenderness, texture and grain, beefy aroma, oiliness, umami flavour, roasted flavour, overall flavour intensity, and thus, explained the greater overall acceptability of these two samples. Lee and Choi [13] showed that the development of marbling results in changes of the collagen content and its solubility, mechanical strength of intramuscular connective tissue, fiber diameter, avoidance of sarcomere shortening, and disorganisation of the perimysia, which account for the improvement in beef tenderness. The author also suggested that beef with a high marbling score should be positioned for higher grade for important sensory attributes (juiciness, tenderness, and flavour-likeness) by consumers.

On the upper left quadrant, KK breed, which has the furthest distance from most sensorial attributes measured, is also far away from IMF. KK, as aforementioned, with the significantly lowest IMF (6.86%) and sensory acceptability, was found to be characterised by attributes including rancid and nutty aroma, beefy and caramelised flavour, internal pink colour, and springy texture. Brahman, on the lower quadrant, somehow does not fall under either sides of the ellipses but closer to the texture and after-taste-related characteristics which include chewy, moderately hard texture, and tooth-packing attributes. These findings are consistent with the instrumental texture WBSF results (Figure 2). The variation in the meat texture quality can be attributed to the origin of the cattle breed. Brahman, which comes from the *Bos indicus* group has a tougher and harder texture as perceived by trained panellists as compared with Wagyu, which belongs to the *Bos taurus* group. According to Leal-Gutiérrez et al [29], the composition of calpastatin is higher than calpain protease enzyme in *Bos indicus* beef as compared with *Bos taurus*. Calpastatin as an inhibitor of calpain protease, contributes to the disturbance of the proteolysis process in softening the muscle tissue of the meat [16,29]. As a result, the meat from *Bos indicus* is tougher than *Bos taurus*. Heat shock protein (HSP) was also reported in *Bos indicus* group which increases the rate of shrinkage process of the protein structure, resulting in the chewy texture of this beef [29].

## CONCLUSION

The cooked ribeye steaks were mainly characterised by the level of IMF. The function of IMF positively influences sensory quality traits as the steaks with high IMF were profiled as tender, juicy, and flavourful. The positive correlation between IMF and overall acceptability has been firmly established by Wagyu and Angus. The presence of negative sensory drivers including bitter flavour, springiness, rancidness, nutty aroma, and compact texture led to the lack of overall acceptability in KK samples. Attention may be given to meat pre-treatment approach and selective method of cooking so that these issues could be minimised.

## Figures and Tables

**Table 1:** The list of the reference foods for reliability test

Reliability test	Tested attributes	Sample	Treatment (%)
1	Sweetness	Table sugar	10
2	Saltiness	Table salt	30
3	Sourness	Vinegar	50

The method used in reliability test was adopted from Meilgaard et al [24].

**Table 2:** Descriptive attributes and definitions used to evaluate cooked ribeye steak

Attribute	Definition and method of evaluation
Appearance	Take off the aluminium foil wrapper and look at the sample.
Surface colour; SC (A) 0, light brown; 15, dark brown	Browning colour intensity belongs to steak muscles after being cooked.
Internal colour; IC (A) 0,Palest pink; 15,Brightest pink	Pink colour intensity in the internal fat of the steak after being cooked.
Texture and grain; TG (A) 0, compact and dense; 15, loose and open	Quantification of tactile firmness in the internal fat and the cooked lean meat of the steak.
Aroma	Take off the aluminium foil wrapper and smell the sample
Sweet 0, none; 15, strong	One of the basic smell. Often considered pleasing while exhibiting characteristics of sugar.
Sour 0, none; 15, strong	One of the basic smell. Often considered sharp, tart and acidic.
Beefy 0, none; 15, strong	Amount of beef flavour identity in the sample
Nutty 0, none; 15, strong	Odour that reminds one of nuts
Rancid 0, none; 15, strong	Intensity of rancid odour perception. An unpleasant aroma or flavour, often produced by oxidized or decomposed oils.
Texture	Touch of the sample with finger and also in the mouth and then compress between tongue and palate
Oiliness 0, none; 15, strong	Amount of shiny moisture-fat coating the surface of the meat.
Springiness 0, no recovery; 15, very springy	Degree that sample returns to original shape after partial compression in the line perception of long and parallel coarse particles in the meat during the touch.
Chewiness 0, low; 15, high	Amount of work required to chew the sample until swallow.
Tenderness 0, tender; 15, hard	Easy to chew. Opposite of toughness or hardness.
Juiciness	The amount of liquid release during chewing
Juiciness 0, dry; 15, moist	Moisture released by the product in the mouth as a result of the initial chewing until final chewing.
Mouthfeel 0, poor; 15, strong	Degree that moisture release by moisture and fat persists or continues during chewing then feeling a physical sensations that determine our enjoyment of food.
Flavour	The intensity of each flavour during chewing
Salty 0, none; 15, strong	One of the basic tastes. Often considered pleasing while exhibiting characteristics of salt.
Sweet 0, none; 15, strong	One of the basic tastes. Often considered pleasing while exhibiting characteristics of sugar.
Sour 0, none; 15, strong	One of the basic tastes. Often considered sharp, tart and acidic.
Bitter 0, none; 15, strong	One of the basic tastes. Often considered harsh and unpleasant in abundance.
Beefy 0, none; 15, strong	Amount of beef flavour identity in the sample.
Roasted 0, none; 15, strong	The smoky flavour note associate with browning process.
Aftertaste	The left over flavour of consumed food sample
Sustained buttery 0, none; 15, strong	Associated with creamy and dense mouthfeel; often evident in products like containing butter or cream and then When a product leaves, a coating on the palate that does not dissolve easily is remained.
Tooth-packing 0, none; 15, strong	Perception of mouth that the meat remains stuck to the teeth once the chewing is finished or mastication done.
Overall acceptability 0, extremely dislike; 15, extremely like	Based on the consumer preference and depends on their choice in every attribute.

**Table 3:** Physical characteristics of ribeye steak from four different cattle breeds

Parameter	Wagyu	Angus	Brahman	Kedah-Kelantan; KK
Intramuscular fat (IMF %)	33.90±1.00^a^	20.87±1.60^b^	12.17±1.63^c^	6.86±0.67^d^
Length of steak (cm)	17.40±0.58^a^	15.57±0.21^b^	10.50±0.10^c^	9.63±0.21^d^
Width of steak (cm)	11.43±0.21^a^	10.43±0.21^b^	13.03±0.21^c^	6.30±0.15^d^
Shear force; raw (N/mm^2^)	5.61±0.33^a^	11.44±0.15^b^	16.33±0.41^c^	15.51±0.05^c^
Shear force; cooked (N/mm^2^)	14.72±0.81^b^	33.30±1.14^a^	43.00±3.90^a^	36.59±7.29^a^

The data was stated in mean±standard error.

a–d Different alphabet in the same row showed significant difference (p<0.05).

**Table 4:** Mean intensity scores of ribeye steak obtained from descriptive analysis of beef samples and their intramuscular fat level

Descriptive analysis	Wagyu	Angus	Brahman	Kedah-Kelantan
Intramuscular fat (%)	33.90±1.00^a^	20.87±1.60^b^	12.17±1.63^c^	6.86±0.67^d^
Appearance (A)				
Surface colour; SC (A) 0, light brown; 15, dark brown	10.56±0.84^ab^	11.49±0.82^a^	8.57±0.84^bc^	7.26±1.07^c^
Internal colour; IC (A) 0, palest pink; 15, brightest pink	4.15±0.82^a^	3.65±0.73^a^	2.90±0.52^a^	4.88±0.74^a^
Texture and Grain; TG (A) 0, compact and dense; 15, loose and open	6.87±0.56^a^	5.98±0.85^ab^	5.60±0.87^ab^	4.46±0.67^b^
Aroma (Aro)				
Sweet; Sw (Aro) 0, none; 15, strong	3.31±1.00^a^	3.53±1.14^a^	3.64±1.12^a^	2.50±0.78^a^
Sour; So (Aro) 0, none; 15, strong	3.02±1.03^a^	2.15±0.88^a^	2.92±0.94^a^	2.07±0.62^a^
Beefy; Beefy (Aro) 0, none; 15, strong	9.92±0.65^a^	9.61±0.82^a^	9.52±0.93^a^	7.74±1.09^a^
Nutty; N(Aro) 0, none; 15, strong	6.24±0.71^a^	6.50±0.99^a^	5.20±0.66^a^	7.34±0.91^a^
Rancid; R(Aro) 0, none; 15,s trong	1.94±0.66^a^	2.41±0.96^a^	1.86±0.69^a^	2.73±0.76^a^
Texture (T)				
Oiliness; oil (T) 0, none; 15, strong	9.76±0.88^a^	8.42±0.84^a^	4.88±1.03^b^	5.02±0.92^b^
Springiness; Sp (T) 0, no recovery; 15, very springy	5.87±0.76^a^	5.67±0.81^a^	6.80±0.67^a^	7.36±0.67^a^
Chewiness; Chw (T) 0, low; 15, high	5.45±0.95^b^	6.69±0.78^ab^	8.26±0.57^a^	7.22±0.52^ab^
Tenderness; T (T) 0, tender; 15, hard	3.45±0.87^b^	7.29±0.67^a^	7.37±0.43^a^	6.67±0.60^a^
Juiciness (J)				
Juiciness; J (J) 0, dry; 15, moist	9.34±0.93^a^	7.80±0.90^ab^	6.00±0.77^b^	6.30±1.03^b^
Mouthfeel; MF (J) 0, poor; 15, strong	9.19±1.14^a^	7.81±1.02^a^	5.99±0.77^b^	6.56±0.93^ab^
Flavour (F)				
Salty; Sa (F) 0, none; 15, strong	3.57±1.13^a^	3.56±1.08^a^	2.25±0.59^a^	2.25±0.77^a^
Sweet; Sw (F) 0, none; 15, strong	3.73±1.34^a^	3.35±1.15^a^	3.43±1.18^a^	2.21±0.68^a^
Sour; So (F) 0, none; 15, strong	2.93±1.03^a^	2.50±1.02^a^	2.77±0.81^a^	1.76±0.61^a^
Bitter; B (F) 0, none; 15, strong	2.36±0.83^b^	3.74±1.04^b^	2.46±0.86^b^	5.04±0.85^a^
Umami; U (F) 0, none; 15, strong	4.55±1.35^a^	4.24±1.32^a^	4.15±1.10^a^	4.26±1.06^a^
Caramel; C (F) 0, none; 15, strong	2.36±1.06^a^	2.92±1.12^a^	2.13±0.93^a^	2.81±1.00^a^
Beefy; Beefy (F) 0, none; 15, strong	9.51±1.02^a^	8.93±0.77^a^	8.04±0.79^a^	7.06±1.01^a^
Roasted; Ro (F) 0, none; 15, strong	8.03±0.71^a^	7.87±0.80^a^	5.27±0.92^b^	7.78±0.74^a^
OFI (F) 0, extremely dislike; 15, extremely like	8.62±1.22^a^	7.14±0.93^ab^	6.44±0.57^ab^	5.00±1.87^b^
After taste (AT)				
Sustained buttery; SB (AT) 0, none; 15, strong	11.65±4.40^a^	5.33±1.09^b^	4.60±0.85^b^	6.00±1.87^b^
Tooth-packing; TP (AT) 0, none; 15, strong	5.75±1.13^a^	6.56±0.89^a^	6.38±0.93^a^	6.38±0.96^a^
Overall acceptance; AO 0, extremely dislike, 15, extremely like	9.95±0.73^a^	7.19±1.06^b^	5.14±0.67^bc^	4.40±0.93^c^

a–d Means with different superscripts in the same row differ (p<0.05).
